# Subretinal macrophages produce classical complement activator C1q leading to the progression of focal retinal degeneration

**DOI:** 10.1186/s13024-018-0278-0

**Published:** 2018-08-20

**Authors:** Haihan Jiao, Matt Rutar, Nilisha Fernando, Ted Yednock, Sethu Sankaranarayanan, Riemke Aggio-Bruce, Jan Provis, Riccardo Natoli

**Affiliations:** 1 The John Curtin School of Medical Research, The Australian National University, Building 131, Garran Rd, Canberra, ACT 2601 Australia; 2 Department of Anatomy and Neuroscience, The University of Melbourne, Parkville, VIC Australia; 3 Annexon Biosciences, South San Francisco, CA USA; 4 ANU Medical School, The Australian National University, ACT, Canberra, Australia

**Keywords:** Macrophages, Microglia, Complement system, Classical pathway, C1q, Inflammasome, Retinal degeneration, Photo-oxidative damage, Inflammation, Age-related macular degeneration

## Abstract

**Background:**

The role of the alternative complement pathway and its mediation by retinal microglia and macrophages, is well-established in the pathogenesis of Age-Related Macular Degeneration (AMD). However, the contribution of the classical complement pathway towards the progression of retinal degenerations is not fully understood, including the role of complement component 1q (C1q) as a critical activator molecule of the classical pathway. Here, we investigated the contribution of C1q to progressive photoreceptor loss and neuroinflammation in retinal degenerations.

**Methods:**

Wild-type (WT), *C1qa* knockout (*C1qa*^*−/−*^) and mice treated with a C1q inhibitor (ANX-M1; Annexon Biosciences), were exposed to photo-oxidative damage (PD) and were observed for progressive lesion development. Retinal function was assessed by electroretinography, followed by histological analyses to assess photoreceptor degeneration. Retinal inflammation was investigated through complement activation, macrophage recruitment and inflammasome expression using western blotting, qPCR and immunofluorescence. C1q was localised in human AMD donor retinas using immunohistochemistry.

**Results:**

PD mice had increased levels of *C1qa* which correlated with increasing photoreceptor cell death and macrophage recruitment. *C1qa*^*−/−*^ mice did not show any differences in photoreceptor loss or inflammation at 7 days compared to WT, however at 14 days after the onset of damage, *C1qa*^*−/−*^ retinas displayed less photoreceptor cell death, reduced microglia/macrophage recruitment to the photoreceptor lesion, and higher visual function. *C1qa*^*−/−*^ mice displayed reduced inflammasome and IL-1β expression in microglia and macrophages in the degenerating retina. Retinal neutralisation of C1q, using an intravitreally-delivered anti-C1q antibody, reduced the progression of retinal degeneration following PD, while systemic delivery had no effect. Finally, retinal C1q was found to be expressed by subretinal microglia/macrophages located in the outer retina of early AMD donor eyes, and in mouse PD retinas.

**Conclusions:**

Our data implicate subretinal macrophages, C1q and the classical pathway in progressive retinal degeneration. We demonstrate a role of local C1q produced by microglia/macrophages as an instigator of inflammasome activation and inflammation. Crucially, we have shown that retinal C1q neutralisation during disease progression may slow retinal atrophy, providing a novel strategy for the treatment of complement-mediated retinal degenerations including AMD.

**Electronic supplementary material:**

The online version of this article (10.1186/s13024-018-0278-0) contains supplementary material, which is available to authorized users.

## Background

The complement cascade comprises three pathways – classical, mannose-binding lectin, and alternative pathway – all of which converge at the proteolysis of complement component 3 (C3), promoting complement activation and downstream assembly of membrane attack complex (MAC) [1]. Activation of the classical pathway is triggered by complement component 1q (C1q) binding to immune complexes on pathogenic cell surfaces, or atypical activators such as modified lipids, apoptotic cells [2], advanced glycation end products [3] and C-reactive protein [4]. C1q binding promotes the proteolysis of C4, C2, C3 and the downstream activation of the complement cascade, propagating the effector functions of complement, including the lysis of target cells [1].

In neurodegenerative diseases such as Age-Related Macular Degeneration (AMD), complement dysregulation as a consequence of environmental pressures and/or gene mutations, is known to contribute to disease progression [5–7]. AMD is the leading cause of blindness in the Western World, posing a significant economic burden with an annual global cost of 350 billion dollars [8]. Atrophic or “dry” late-stage AMD is characterised by the emergence of geographic atrophy, in which a central retinal lesion in the photoreceptors and retinal pigment epithelium (RPE) develops and progressively expands over time [8]. Genome-wide association studies (GWAS) indicate a link between dysregulation of multiple complement pathways (e.g. C3, C4, C1s, CFI, SERPING1) and the progressive expansion of the macular lesion in geographic atrophy [9]. In addition, histopathological investigations show a range of complement components and factors from all pathways present in drusen of AMD donor eyes (reviewed in [7]). These drusen components are associated with the accrual of activated microglia and infiltrating blood-borne macrophages in the outer retina, which is a well-established feature of AMD pathogenesis [10].

Our previous studies have shown upregulation of a range of complement pathway genes, including *Cfb*, *C3* and *Cfh* after photo-oxidative retinal degeneration, and demonstrate a critical role for C3-expressing retinal macrophages [11, 12]. However, the contribution of each complement pathway to C3 activation is still largely unknown, impeding the development of effective anti-complement drugs for retinal degenerations [13, 14]. Other investigators have utilised *Cfb*^*−/−*^, *C1qa* ^*−/−*^ and *Mbl*^*−/−*^ mice to demonstrate the involvement of all complement pathways to experimental choroidal neovascularization [15]. Despite strong evidence supporting the involvement of the alternative pathway in retinal degenerations [16], it has been suggested that the alternative pathway is not the only contributor towards retinal degenerations, and that other complement pathways may also facilitate disease progression [15].

Emerging evidence has implicated the classical pathway in AMD, including genetic polymorphisms [17] and an abundance of autoantibodies associated with AMD [18–20]. C1q, the activator molecule of the classical complement pathway, has also been implicated in a number of complement-independent processes including activation of the inflammasome, another key component of the innate immune system [21], and by acting as a bridging molecule between the innate and adaptive immune system [22, 23]. Recently, C1q was found to promote microglial activation and degeneration in retinal ischemia/reperfusion (I/R) injury [24]. However, the role of C1q in the progression of retinal degenerations remains unclear. In this study, we aimed to elucidate the contribution of the classical complement pathway to disease progression, using a model of photo-oxidative damage (PD), in which complement activation by microglia and macrophages is a key feature leading to progressive photoreceptor loss [11]. Our findings illustrate the role of the classical complement pathway in focal retinal degeneration, showing subretinal macrophages as key players in delivering C1q, and that blocking C1q activity during degeneration may be a useful strategy to slow the progression of retinal atrophy.

## Methods

### Animal experimentation

All procedures were performed in accordance with the Association for Research in Vision and Ophthalmology (ARVO) Statement for Use of Animals in Ophthalmic and Vision Research. The study was approved by the Australian National University Animal Experimentation Ethics Committee (Ethics ID: A2014/56 and A2017/41). Complement component 1, q subcomponent, alpha polypeptide knock out (*C1qa*^*−/−*^) mice were used for this study (C1qa < tm1Mjw>). C57BL/6 J mice were used as wild-type (WT) controls. Animals were born and raised in dim (5 lx) cyclic light conditions in 12:12 h cycle, and house in individual vented cages (IVCs). Food and water was provided in constant supply and cages changed on a weekly basis. Adult mice aged between postnatal day (P) 60-90 were used for all experiments. Equal numbers of male and female mice were used throughout the study to avoid any gender biases. Animals with no exposure to photo-oxidative damage were used as dim-reared controls.

To induce photo-oxidative damage (PD), mice were housed in Perspex boxes coated with a reflective interior, and exposed to 100Klux of natural white LED for 1, 3, 5 and 7 days, with free access to food and water, as described in our previous methodology [25]. Some animals were recovered in dim light conditions and were collected at 8, 10 and 14 days following the onset of PD. Each animal was administered with pupil dilator eye-drops twice daily during PD (Atropine Sulphate 1%*w*/*v* eye-drops; Bausch and Lomb, NSW, Australia). Electroretinography (ERG) was used to measure mouse retinal function in response to full-field flash stimuli under scotopic conditions as described previously [25]. Animals were euthanized with CO_2_ prior to tissue collection. Eyes were collected and processed for cryosections or RNA extraction, as previously described in our publications [12, 25].

### C1q neutralisation using ANX-M1

C1q neutralisation strategies included local pre-treatment, local post-treatment and systemic treatment following the paradigms listed below. Briefly, a neutralising antibody to C1q, ANX-M1 and a non-specific isotype control IgG monoclonal antibody (mAb) were provided by Annexon Biosciences (CA, USA). Monoclonal antibody M1 binds and neutralises C1q thereby preventing the activation of the classical complement pathway. ANX-M1 anti-C1q antibody or an IgG isotype control antibody were formulated in endotoxin-free 0.1 M phosphate-buffered saline (Thermo Fisher Scientific, MA, USA). All experiments were performed on adult C57BL/6 J mice under double-blind conditions. Intravitreal injections were performed as described in our previous publication, with a volume of 1 μl injected into each mouse eye [26].

### Local inhibition

#### Pre-treatment

A 1 μl solution containing either the ANX-M1 anti-C1q antibody or an IgG isotype control antibody (7.5 μg/μl) was injected into each mouse eye, prior to the commencement of photo-oxidative damage (day 0). One animal from each cohort (ANX-M1 anti-C1q antibody and IgG control) were placed in the same light box for 7 days of photo-oxidative damage. Retinas were assessed at 7 days after photo-oxidative damage (day 14) and analysed (*N* = 10/group).

### Local inhibition

#### Post-treatment

A 1 μl solution of ANX-M1 anti-C1q antibody or IgG isotype control antibody (7.5 μg/μl) was administered intravitreally immediately after completion of the 7-day photo-oxidative damage procedure (day 7) and placed in dim-cyclic light until day 14, for retinal assessment (*N* = 10/group).

### Systemic inhibition

100 mg/kg of ANX-M1 anti-C1q antibody or an IgG isotype control antibody was administered intraperitoneally on day 0 (prior to the PD procedure), day 4 (during the PD procedure) and on day 8 (the day after completion of the PD procedure) in order to sustain complement inhibition. Hemolytic assay and retinal assessment were performed at day 12 (N = 10/group) as per our previous publications [11, 27].

### TUNEL and immunohistochemistry

A terminal deoxynucleotidyl transferase (TdT) dUTP nick-end labelling (TUNEL) assay (Roche Diagnostics, Basel, Switzerland) was used to detect photoreceptor cell death. Retinal cryosections were used for the TUNEL assay according to our previously described protocols, with minor modifications [28, 29]. Sections were counterstained with the DNA-specific dye bisbenzimide (1:10000; Sigma Aldrich, MO, USA) for visualisation of cell nuclei.

To detect and localise specific proteins, immunohistochemistry was performed on retinal cryosections according to previously published methodology, with minor modifications [12, 30]. A list of primary antibodies is provided in Table 1.

**Table 1:** Primary antibodies used for immunohistochemistry and Western blot

Antibody	Target	Dilution	Source	Catalog #
Rabbit α-IBA1	Ionised calcium binding protein 1	1:500	Wako Chemicals	019-19,741
Goat α-IL-1β	IL-1 beta/IL1F2	1:1000	R&D Systems	AF-501
Goat α-C3d	Complement component C3d	1:2000	R&D Systems	AF-2655
Rabbit α-C1q	C1q, clone 4.8	1:400	Abcam	AB182451
Rabbit α-C1q	C1q complement isolated from human plasma	1:1000	Dako	F0254
Rabbit α-NALP3/NLRP3	1-50 amino acids of human NLRP3	1:500	Novus Biologicals	NPB2-12446SS
Rat α-F4/80	F4/80 clone A3-1	1:100	Abcam	AB6640
Rabbit α-GAPDH	GAPDH	1:4000	Sigma Aldrich	G9545

### Fluorescence analysis and imaging

TUNEL^+^ and IBA1^+^ cells were quantified along the full length of retinal cryosection in duplicate sections per animal. For photoreceptor cell death, only TUNEL^+^ cells in the ONL were counted. IBA1^+^ cells in the outer retina (including subretinal space, ONL-RPE) were taken across the cryosection. For each section, number of TUNEL^+^ or IBA1^+^ cells was recorded (superior to inferior) in increments of 0.5 mm across the full length of retinal cryosection. Total cell counts were averaged from at least 2 sections per animal with 6 animals for each experimental group.

To quantify photoreceptor loss in retinal cryosections, the number of rows of photoreceptor nuclei was counted within the retinal lesion site (superior retina, 1 mm away from the optic nerve head). Five measurements were taken per retina in duplicate per animal.

Fluorescence in retinal cryosections was visualised and imaged using a laser-scanning A1^+^ confocal microscope (Nikon, Tokyo, Japan). Images were acquired using NIS-Elements AR software (Nikon). Negative control slides (no primary antibody) were imaged with each immunolabelled slide to determine specificity of staining and set up the threshold for fluorescence intensity for comparison to positively-stained slides. Images were processed and assembled into panels using Photoshop CS6 software (Adobe Systems, CA, USA).

### Fluorescence-activated cell sorting (FACS)

Mouse retinal microglia and macrophages (CD11b^+^) were isolated using a fluorescence-activated cell sorter (BD FACSAria II; BD Biosciences, NJ, USA), using previously described protocols with minor modifications [25, 30, 31]. Mouse retinas were pooled from 2 animals (4 retinas) for each sample. Cells were stained using an anti-mouse CD11b-Phycoerythrin (PE) conjugated antibody (#101207, 1:500; Biolegend, CA, USA). FACS-isolated cells were then used immediately for RNA extraction.

### Quantitative real-time polymerase chain reaction (qPCR)

RNA extraction and purification was performed on retinas and isolated CD11b^+^ cells using a combination of TRIzol reagent (Thermo Fisher Scientific) and an RNAqueous Total RNA Isolation Kit (Thermo Fisher Scientific) as described in our previous publication [32]. cDNA was prepared from 500 ng of each RNA sample using a Tetro cDNA Synthesis Kit (Bioline Reagents, London, UK) according to the manufacturer’s protocol.

Gene expression changes were measured via qPCR using Taqman hydrolysis probes (listed in Table 2) and Taqman Gene Expression Master Mix (Thermo Fisher Scientific). Each qPCR was run using a QuantStudio 12 K Flex instrument (Thermo Fisher Scientific) at the Biomolecular Resource Facility (JCSMR, ANU). Analysis was performed using the comparative cycle threshold method (ΔΔC_t_) which was normalised to the expression of *Gapdh* and *Actb* reference genes, as established previously [33, 34].

**Table 2:** Taqman hydrolysis probes used for qPCR (Thermo Fisher Scientific)

Gene symbol	Name	Accession #	Catalog #
Actb	Actin-beta	AK078935	Mm01205647_g1
C1qa	Component 1, q subcomponent, alpha polypeptide	NM_007572.2	Mm00432142_m1
C2	Component 2	NM_013484	Mm00442726_m1
C4	Component 4	NM_011413	Mm01132415_g1
Casp-1	Caspase-1	NM_009807	Mm00438023_m1
Casp-8	Caspase-8	NM_001080126	Mm01255716_m1
Cfb	Factor B	NM_001142706.1	Mm00433918_g1
Cfh	Factor H	NM_009888	Mm01299248_m1
Cntf	Ciliary neurotrophic factor	NM_170786.2	Mm00446373_m1
Fgf2	Fibroblast growth factor 2	NM_008006.2	Mm01285715_m1
Gapdh	Glyceraldehyde-3-phosphate dehydrogenase	NM_001289726	Mm99999915_g1
Gfap	Glial fibrillary acid protein	NM_010277.3	Mm01253033_m1
Il-18	Interleukin 18	NM_008360.1	Mm00434226_m1
Il-1b	Interleukin 1-beta	NM_008361	Mm00434228_m1
Nlrp3	NLR Pyrin Domain Family 3	NM_145827	Mm00840904_m1
Serping1	Serine peptidase inhibitor member 1	NM_009776	Mm00437834_m1

### Western blot

Retinas were collected into Cellytic M buffer (Sigma-Aldrich) containing a Protease Inhibitor Cocktail (Sigma-Aldrich). Western blotting was performed on retinal protein lysates according to previously described methods with minor modifications [35, 36]. 20 μg of denatured protein was loaded onto a 4-20% Mini-Protean TGX Precast Protein gel (Bio-Rad, CA, USA) followed by semi-dry transfer to a nitrocellulose membrane. A list of primary antibodies used for Western blot are provided in Table 1. A secondary antibody-peroxidase conjugate was used for visualisation (Bio-Rad). The protein was visualised with chemiluminescence using a Clarity Western ECL kit (Bio-Rad) and images were captured and analysed using a Chemidoc MP with Image Lab software (Bio-Rad). The expression of the protein of interest was normalised to GAPDH.

### Statistical analysis

All statistical analysis was performed using Prism 6 (GraphPad Software, CA, USA). Unpaired student t-test, one-way analysis of variance (ANOVA) or two-way ANOVA were run as appropriate for each dataset. Multiple comparisons were run with appropriate post-hoc tests to determine statistical significance for the interactions between each experimental group (*P* < 0.05). Graphs are displayed as mean ± SEM.

## Results

### *C1qa* gene expression following photo-oxidative damage

**Fig. 1:**
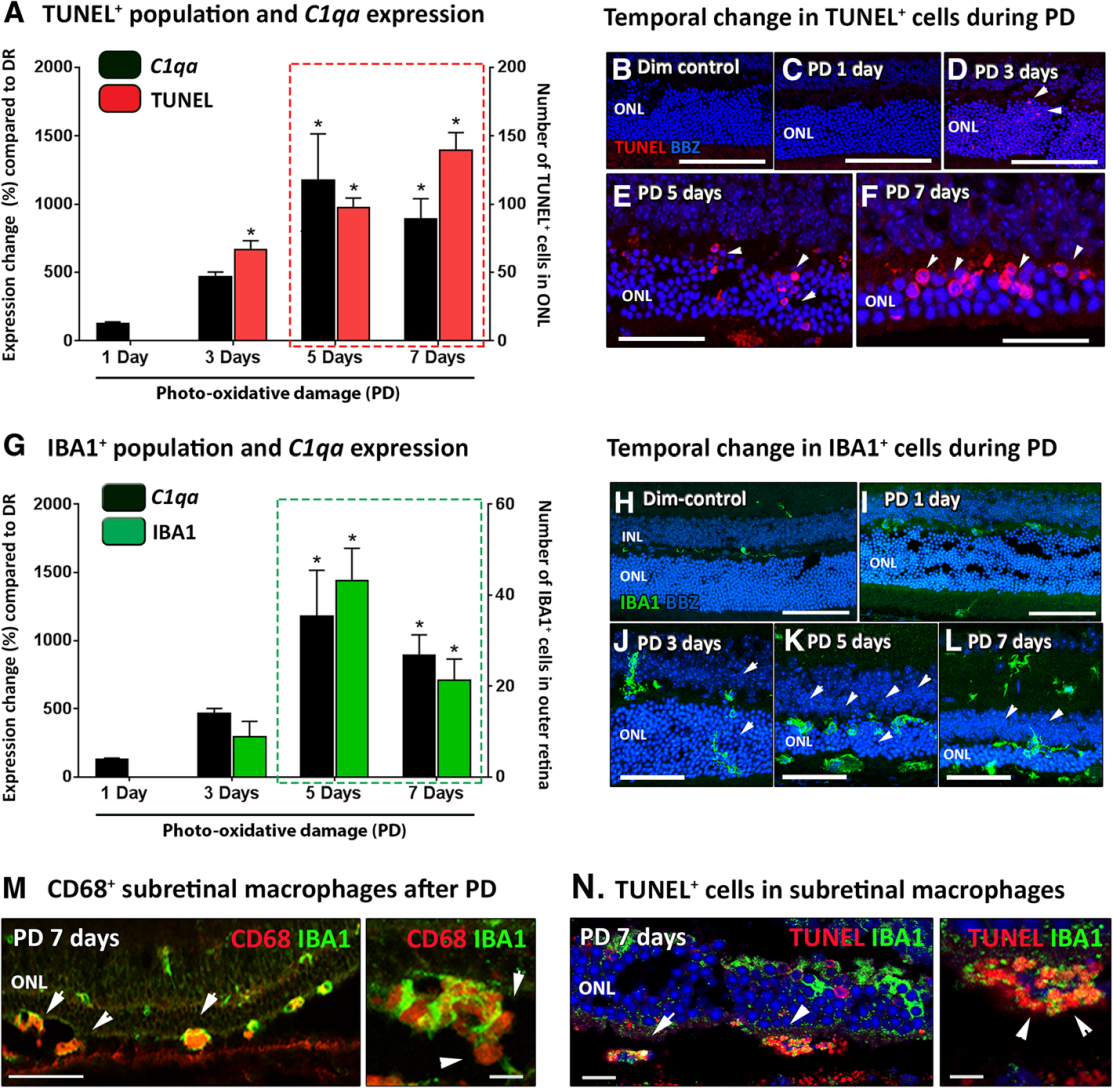
*C1qa* gene expression following days of photo-oxidative damage (PD) in wild-type animals (WT). **a** Increasing numbers of TUNEL^+^ cells in the outer retina was temporally associated with changes in expression of *C1qa*. **b**-**f** TUNEL^+^ cells (red; arrows) were most abundant in the ONL at 5 (**e**) and 7 days (**f**), and absent in dim-control (**b**). **g** Increasing number of IBA1^+^ macrophages in the outer retina was significant at 5 and 7 days of PD, and overlapped with upregulation of *C1qa* (*P* < 0.05). **h**-**l** IBA1^+^ cells were prominent in retinas at 5 (**k**) and 7 (**l**) days of PD and included the activated/amoeboid cells in outer retina and subretinal space. **m** CD68 immunoreactivity was abundant in IBA1^+^ macrophages located in the subretinal space after 7 days of PD (arrows). **n** TUNEL and IBA1 double-labelling displayed that IBA1^+^ macrophages contained multiple TUNEL^+^ apoptotic cells in the subretinal space after PD (arrows). Statistical significance was determined by student t-test and two-way ANOVA accompanied with post-hoc multiple comparison (*N* = 5-6 per group, *represents *P* < 0.05). ONL, outer nuclear layer; INL, inner nuclear layer. For all images, scale bars represent 50 μm

The expression of *C1qa* in the murine retina was studied across the time course of photo-oxidative damage (1, 3, 5 and 7 days of photo-oxidative damage) in wild-type (WT) mice using qPCR (Fig. 1). Retinal *C1qa* gene expression was upregulated in the retina during the course of photo-oxidative damage, compared to dim-reared control retinas (Fig. 1a). *C1qa* expression was significantly elevated at 5 and 7 days (*P* < 0.05, Fig. 1a), but not at 1 or 3 days of photo-oxidative damage. Temporal expression of *C1qa* in the retina coincided with the focal loss of photoreceptors (TUNEL^+^ cells) in the ONL (Fig. 1b-f, arrows). TUNEL^+^ photoreceptor cell death reached peak numbers at 7 days of photo-oxidative damage (*P* < 0.05, Fig. 1a, f). Temporal expression of retinal *C1qa* also correlated with IBA1^+^ microglia/macrophage recruitment to the ONL and subretinal space of the outer retina, peaking at 5 days photo-oxidative damage (Fig. 1g-l), with significance detected at both 5 and 7 days for both *C1qa* expression and IBA1^+^ cell recruitment (*P* < 0.05, Fig. 1g-l). Double-labelling with CD68, a lysosomal protein, demonstrated a phagocytic phenotype of IBA1^+^ macrophages in the subretinal space after photo-oxidative damage (Fig. 1m). After 7 days of photo-oxidative damage, the IBA1^+^ macrophages were highly immunoreactive for CD68; the majority of the subretinal population contained multiple CD68^+^ cellular compartments (Fig. 1m). Double-labelling of TUNEL and IBA1 demonstrated that IBA1^+^ macrophages were in close proximity and direct contact with multiple TUNEL^+^ apoptotic cells in the subretinal space and ONL at 7 days of photo-oxidative damage (Fig. 1n).

### Effect of *C1qa* knockout on the retina

Classical complement pathway knockout mice (*C1qa*^*−/−*^) and WT were exposed to photo-oxidative damage to assess independently the functional significance of *C1qa* (classical pathway) on retinal degeneration. To control for any underlying effects, we thoroughly assessed *C1qa*^−/−^ mice raised in low light conditions for any histopathological or physiological changes, compared to WT (Additional file 1: Figure S1). There were no significant differences in the amplitude of a-wave, b-wave and cone responses in the *C1qa*^*−/−*^ mice compared to WT. There was no significant change in the expression of neurotrophic factor *Cntf* and the stress marker *Gfap*. A reduction in expression was detected in *Fgf2* in *C1qa*^*−/−*^ retinas (*P* < 0.05, Additional file 1: Figure S1).

### *C1qa*^*−/−*^ after 7 days of photo-oxidative damage

**Fig. 2:**
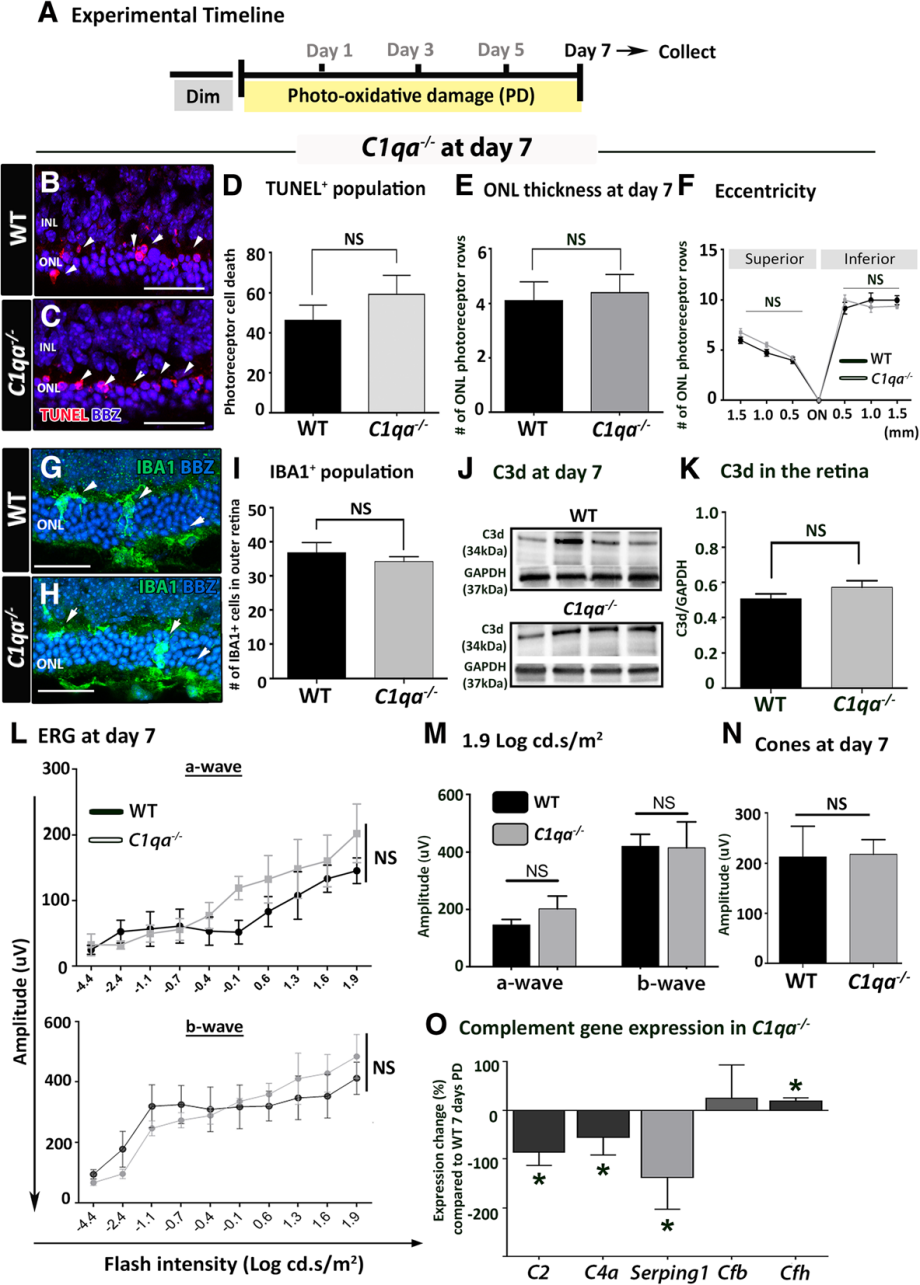
Retinal morphology and measures of inflammation in *C1qa*^*−/−*^ retinas at 7 days of photo-oxidative damage (PD) **(a)**. **b**-**c** Photoreceptor cell death indicated using TUNEL at the focal lesion site in wild-type (WT) and *C1qa*^*−/−*^ retinas. **d**-**f** Quantitative analyses showed no significant difference between *C1qa*^*−/−*^ retinas and WT retinas in the total numbers of TUNEL^+^ cells (**d**), thickness of the outer nuclear layer (ONL) at the lesion site (**e**), or in adjacent retina (**f**) (NS, *P* > 0.05). **g**-**i** The IBA1^+^ cells were shown in amoeboid morphology in the outer retina in WT and *C1qa*^*−/−*^ retinas (arrows, **g**, **h**). There was no remarkable difference in the numbers of IBA1^+^ cells present after 7 days of PD (**i**) (NS, *P* > 0.05). **j**-**k** Abundance of C3d proteins by Western blotting appeared comparable in the two groups (**j**), and confirmed by optical densitometry (**k**). **l**-**n** No significant differences in retinal function were detected in *C1qa*^*−/−*^ compared to WT at 7 days of PD. **o** Expression of classical complement genes *C2, C4a,* and *Serping1* significantly reduced in *C1qa*^*−/−*^ retinas compared to WT (*P* < 0.05), whereas the *Cfh* of the alternative pathway displayed a significant upregulation (*P* < 0.05), but had no significant change in *Cfb* (*P* > 0.05). Student t-test, one- and two-way ANOVA followed by post-hoc multiple comparison were used for analysis (*N* = 6 per group, *represent *P* < 0.05; NS represents no significance). For representative images, scale bars represent 50 μm

After 7 days of photo-oxidative damage similar levels of photoreceptor death, indicated by numbers of TUNEL^+^ cells, were observed in *C1qa*^−/−^ and WT retinas (*P* > 0.05, Fig. 2b-d). Measures of retinal morphologies including thickness of the photoreceptor layer at the focal lesion spot and towards the lesion edge were comparable (*P* > 0.05, Fig. 2e-f). Numbers of IBA1^+^ macrophages found in the outer retina of *C1qa*^*−/−*^ and WT animals were also comparable following 7 days of photo-oxidative damage (*P* > 0.05, Fig. 2g-i). Western blots for complement C3d protein showed an increase in retinal C3d protein following 7 days of photo-oxidative damage compared to dim-reared controls (*P* < 0.05, Additional file 2: Figure S2). However, we showed no difference in the abundance of C3d protein in *C1qa*^*−/−*^ retina and WT at 7 days of photo-oxidative damage (Fig. 2j); C3d protein expression, normalised to the loading control, did not significantly change in *C1qa*^*−/−*^ and WT (*P* > 0.05, Fig. 2k).

ERG analysis showed no differences in a-wave, b-wave, or cone responses in *C1qa*^−/−^ mice compared to WT (*P* > 0.05, Fig. 2l-n). Expression of complement genes in the classical pathway (*C2, C4a, Serping1*), however, were significantly lower in *C1qa*^*−/−*^ retinas compared to WT (*P* < 0.05, Fig. 2o). *Cfh* expression (alternative pathway) was significantly elevated in *C1qa*^*−/−*^ retinas at 7 days of photo-oxidative damage (*P* < 0.05, Fig. 2o), however, there was no significant change in the expression of *Cfb*. Using a photo-oxidative damage model, we have previously shown that these complement genes are upregulated in the retina following photo-oxidative damage compared to dim-reared controls [11].

### The effect of *C1qa* knockout: Extended time course

**Fig. 3:**
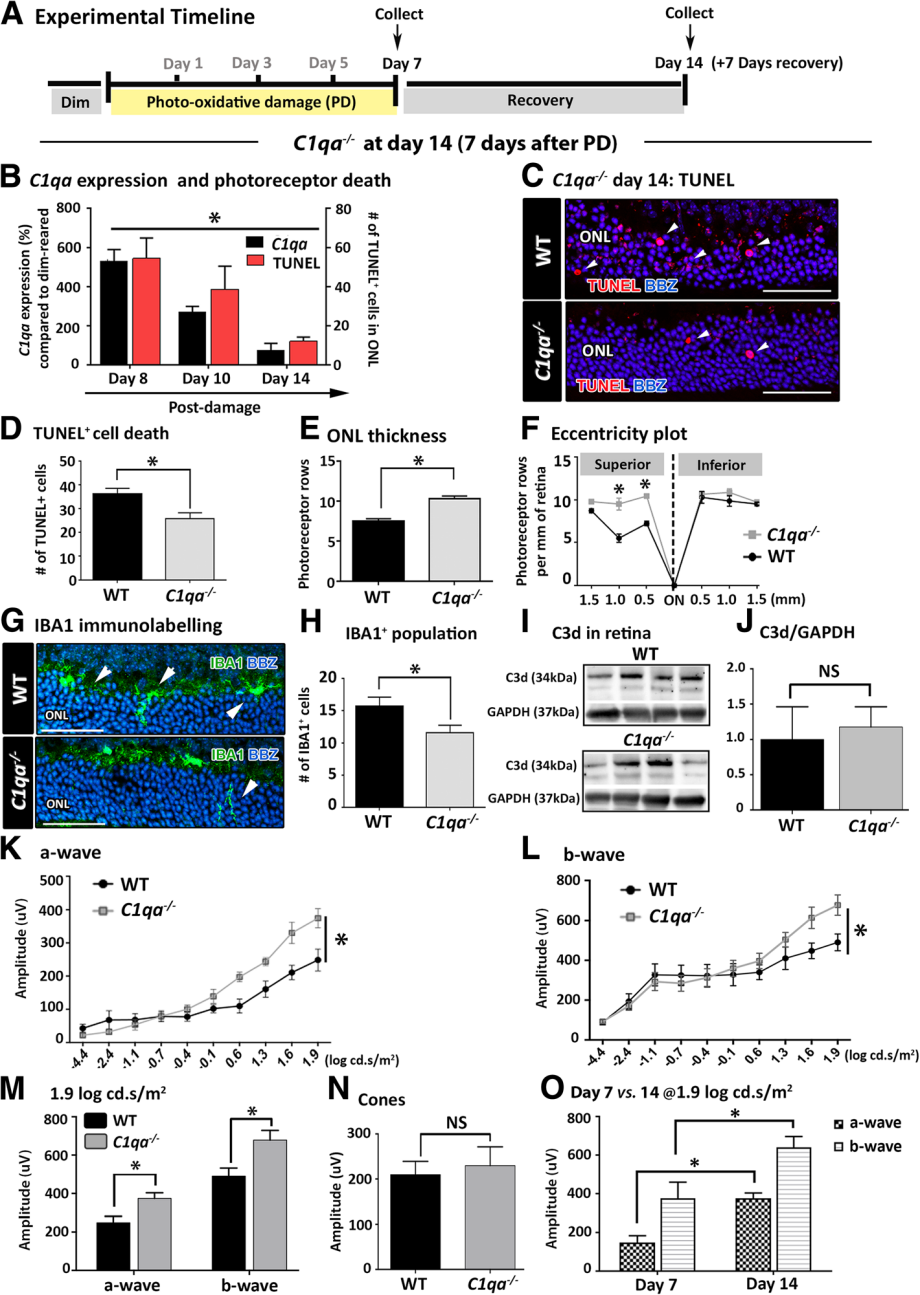
Wild-type (WT) and *C1qa*^*−/−*^ retina at day 14 (7 days after the end of photo-oxidative damage, PD) **(a)**. **b** *C1qa* expression was significantly elevated in WT PD animals compared to WT dim-reared controls between day 8 and day 14, and the expression level decreased across the days of recovery, in concert with TUNEL^+^ photoreceptor cell death (*P* < 0.05). **c**-**d** TUNEL^+^ cells were present in the outer nuclear layer (ONL) of *C1qa*^*−/−*^ and WT retinas, although the rate of cell death was significantly lower in *C1qa*^*−/−*^ retinas (*P* < 0.05). **e**-**f** Photoreceptor row counts were significantly higher in *C1qa*^*−/−*^ compared to WT, in the superior retina (0.5 mm-1.0 mm away from the optic nerve) (*P* < 0.05). **g**-**h** Fewer IBA1^+^ cells were found in the ONL of *C1qa*^*−/−*^ retinas compared to WT, and was statistically significant (*P* < 0.05). **i**-**j** Western blots showed that there was no significant difference in the relative intensity of C3d proteins between *C1qa*^*−/−*^ and WT retinas (*P >* 0.05). **k**-**l** There was a stronger a-wave and b-wave responses in *C1qa*^*−/−*^ animals compared to WT, particularly at higher flash intensities (*P* < 0.05). **m** At the highest flash intensity, *C1qa*^*−/−*^ animals had significantly higher a-wave and b-wave amplitudes compared to WT (*P* < 0.05). **n** Cone responses were not significantly different in *C1qa*^*−/−*^ and WT animals (*P >* 0.05). **o** Comparison of a-wave and b-wave responses at day 7 and 14 showed significantly higher amplitudes in *C1qa*^*−/−*^ retina at day 14. Statistical significance was measured using student t-test and two-way ANOVA followed by post-hoc multiple comparison (*N =* 6 per group; * represents *P* < 0.05; NS represents no significance). For representative images, scale bars represent 50 μm

To assess an effect of the classical pathway on photoreceptor damage beyond the standard 7-day photo-oxidative damage time course, the impact of *C1qa* gene ablation over an extended period *after* photo-oxidative damage was determined (days 8-14, Fig. 3a). Retinal expression of *C1qa* was significantly elevated in WT mice between day 8 (one day after the end of photo-oxidative damage) and day 14 (*P* < 0.05, Fig. 3b) and accompanied by elevated levels of TUNEL^+^ photoreceptor cell death (*P* < 0.05*,* Fig. 3b)*.* Photoreceptor cell death was significantly reduced in the *C1qa*^*−/−*^ compared to WT at day 14 (*P* < 0.05, Fig. 3c, d), and the ONL was significantly thicker (*P* < 0.05, Fig. 3e). The ONL of the superior retina in *C1qa*^*−/−*^ mice was better preserved at the lesion site (0.5 mm) and at 1.0 mm away from the optic disc at day 14, indicated by the presence of significantly more rows of photoreceptors in the ONL compared to WT (*P* < 0.05, Fig. 3f).

The retinal macrophage response in *C1qa*^*−/−*^ mice was attenuated at day 14 (Fig. 3g-h). Fewer IBA1^+^ macrophages were present in the ONL of *C1qa*^*−/−*^ at day 14 compared to WT (Fig. 3g), and the numbers of IBA1^+^ macrophages infiltrating the ONL and subretinal space were significantly less than WT (*P* < 0.05, Fig. 3h). Western blots of complement C3d in *C1qa*^*−/−*^ and WT retina showed no difference in the abundance of complement C3d deposition in *C1qa*^*−/−*^ retina compared to WT at day 14 (*P* > 0.05, Fig. 3i-j).

ERG analyses showed clear differences in retinal function at day 14 between *C1qa*^*−/−*^ and WT (*P* < 0.05, Fig. 3k-n). The a-wave response and b-wave response amplitudes to flash intensities ≥0.6 log cd.s/m^2^ were significantly greater in *C1qa*^*−/−*^ retina compared to WT (*P* < 0.05, Fig. 3k, l). Differences in ERG response characteristics between *C1qa*^*−/−*^ and WT were significant across increasing flash intensities, with the most pronounced differences observed at 1.9 log cd.s/m^2^ (*P* < 0.05, Fig. 3m). There were no differences in the cone responses between the two groups (*P* > 0.05, Fig. 3n). Comparison of ERG responses at day 7 of photo-oxidative damage and day 14 (7 days after photo-oxidative damage) indicate improved retinal function in *C1qa*^*−/−*^ animals at 14 days (*P* < 0.05, Fig. 3o). At day 14, *C1qa*^−/−^ retina had significantly better a-wave responses (~ 100 μV) and b-wave responses (~ 200 μV) than WT at 1.9 log cd.s/m^2^ (*P* < 0.05, Fig. 3o). At day 14, even at low flash intensities, the a-wave amplitudes in *C1qa*^−/−^ retinas were significantly greater than in WT retinas (Fig. 3o).

### Intravitreal C1q inhibition using ANX-M1 anti-C1q antibody

**Fig. 4:**
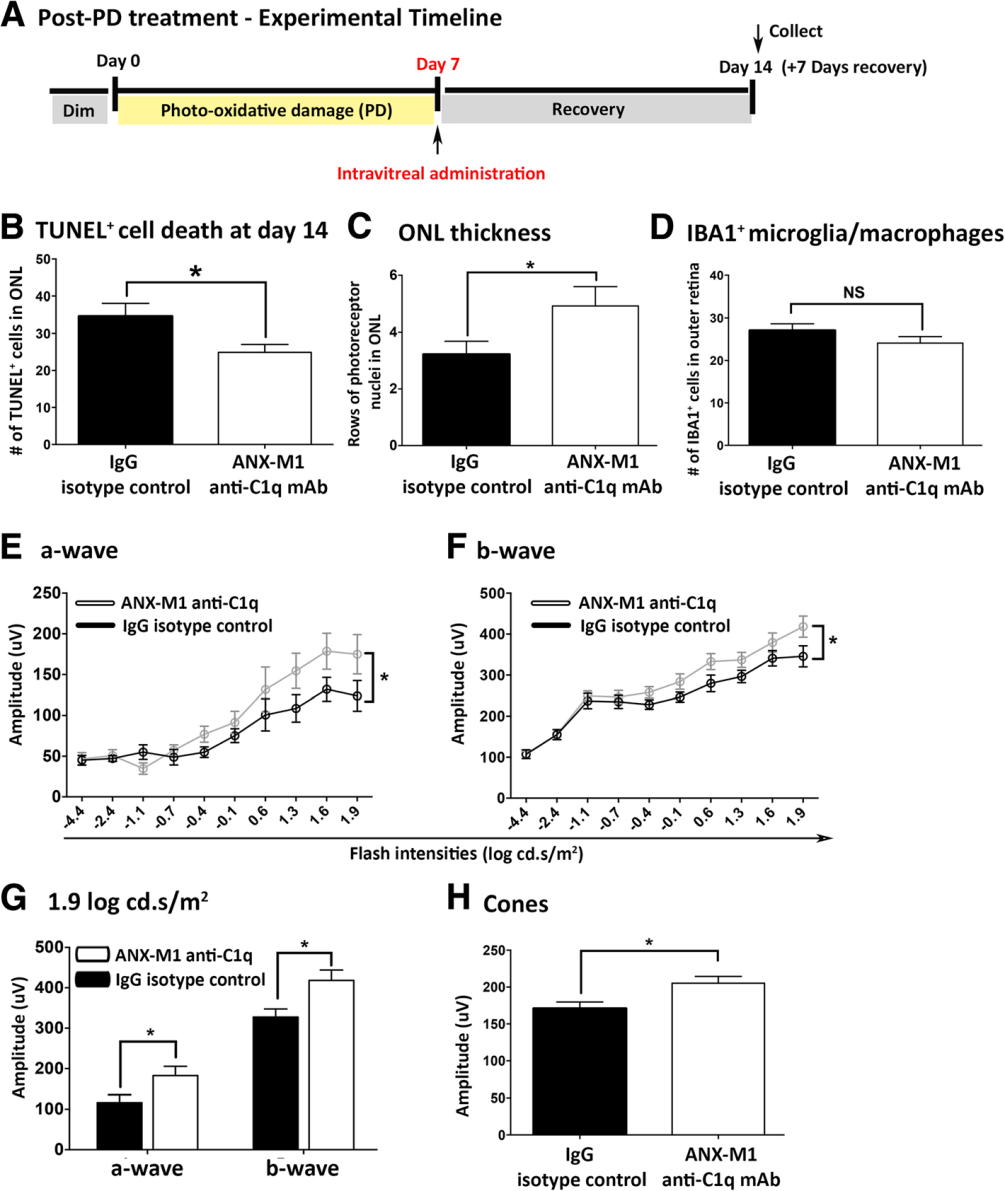
Post-treatment with local neutralisation of C1q and assessment of the retina at day 14 **(a)**. **b** Post-treatment with anti-C1q monoclonal antibody (mAb), ANX-M1, immediately after 7 days of photo-oxidative damage (PD, day 7) reduced the number of TUNEL^+^ cells at 7 days of post damage (day 14), versus IgG control at day 14 (*P* < 0.05). **c** There was a significant reduction in photoreceptor loss in mice treated with ANX-M1 anti-C1q antibody (*P* < 0.05). **d** There was minimal effect on the IBA1^+^ microglia/macrophages in ANX-M1 anti-C1q antibody cohort compared to IgG control antibody (*P* > 0.05). **e-g** Post-treatment of retina with ANX-M1 anti-C1q antibody displayed a larger a-wave (**e**) and b-wave (**f**) compared to IgG control cohort, which was most pronounced at 1.9 log cd.s/m^2^ (**g**), indicating a significantly improved retinal function (*P* < 0.05). **h** Cone response demonstrated a significant improvement in ANX-M1 anti-C1q antibody cohort, compared to IgG control (*P* < 0.05). Statistical analyses were determined by an unpaired student t-test or two-way ANOVA followed by post-hoc multiple comparison (* denotes *P* < 0.05; NS denotes no significance)

To determine whether local inhibition of C1 activation could mitigate the progression in retinal atrophy, we used an antibody against C1q (anti-C1q; ANX-M1, Annexon Biosciences), to block the classical complement cascade. Intravitreal ANX-M1, but not its immunoglobulin G (IgG) isotype control, administered immediately after the 7-day photo-oxidative damage procedure (day 7), protected the photoreceptors against continuous degeneration at day 14 (Fig. 4). Post-damage, ANX-M1 significantly reduced the number of TUNEL^+^ cells in retinas at day 14, compared to IgG control antibody (*P* < 0.05; Fig. 4b). Comparison of retinal thickness showed that the ONL was significantly thicker (*P* < 0.05; Fig. 4c) in the ANX-M1 group. Negligible effects on the number of IBA1^+^ microglia/macrophages in the outer retinal space and ONL were observed in mice treated with anti-C1q antibody, compared to IgG control (*P* > 0.05; Fig. 4d). ERG analyses showed that the response characteristics for rod a-wave and b-wave were significantly better in the ANX-M1 treated animals (*P* < 0.05; Fig. 4e, f), with the ANX-M1 cohort demonstrating significantly higher amplitudes (a- and b-wave) at the highest flash intensities compared to IgG controls (*P* < 0.05; Fig. 4g). Cone responses were also significantly higher in the post-treatment ANX-M1 groups (*P* < 0.05; Fig. 4h).

**Fig. 5:**
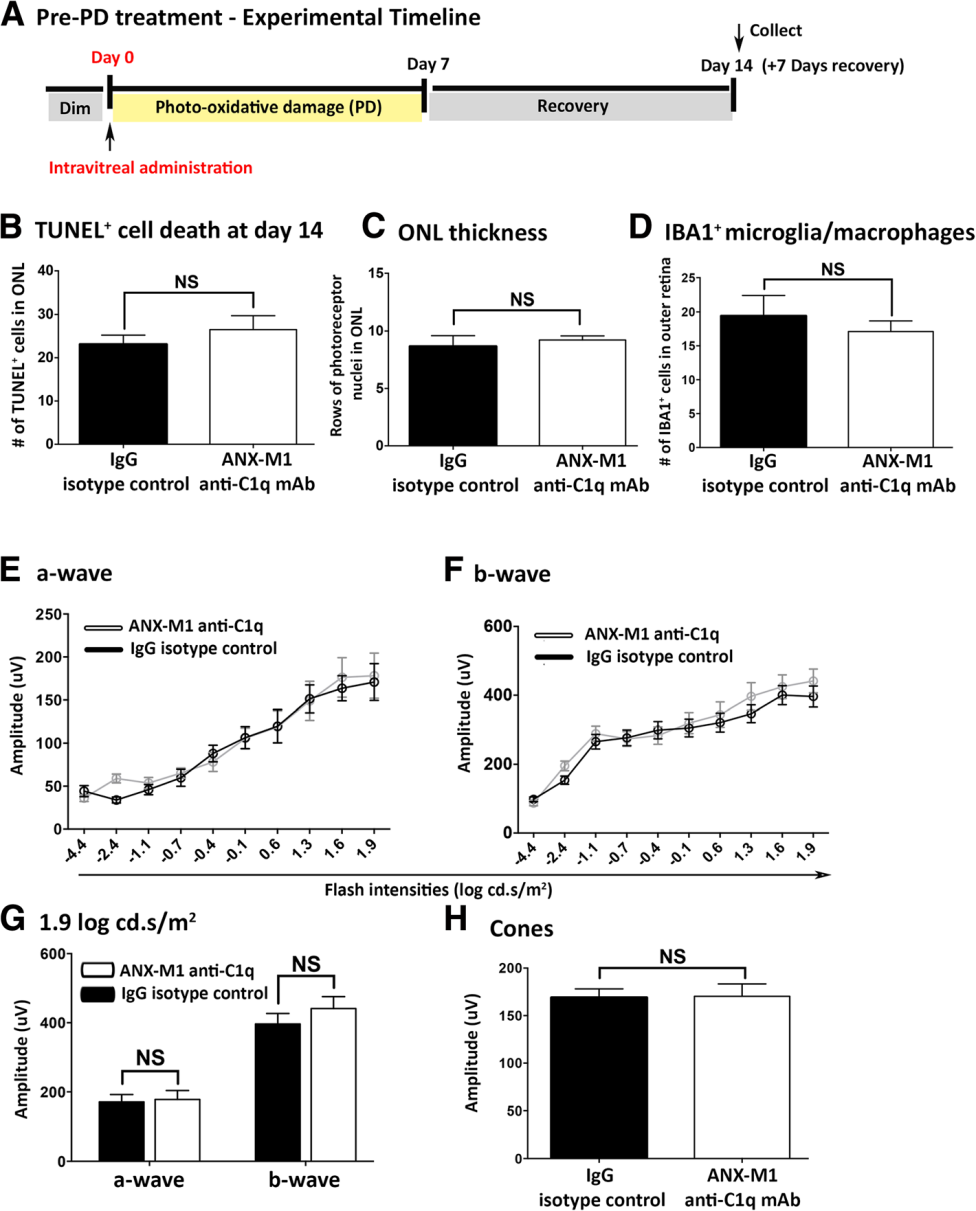
Pre-treatment with local neutralisation of C1q and assessment of retina at day 14 (a). **b** Pre-treatment with ANX-M1 anti-C1q antibody prior to the start of photo-oxidative damage (PD, day 0) showed no significant change in the number of TUNEL^+^ cells at day 14, versus IgG control (*P* > 0.05). **c** There was no difference in outer nuclear layer (ONL) thickness observed between ANX-M1 anti-C1q antibody and IgG control (*P* > 0.05). **d** Minimal effect was observed in the number of IBA1^+^ microglia/macrophages between two cohorts (*P* > 0.05). **e**-**g** Pre-treatment with local inhibition of classical complement did not decrease the retinal function as observed in a-wave **(e)**, b-wave response characteristics **(f)** and mean amplitudes at 1.9 log cd.s/m^2^ (**g)** (*P* > 0.05). **h** No change in the cone response was also observed between mice treated with ANX-M1 anti-C1q antibody and IgG control (*P* > 0.05). Statistical analyses were determined by an unpaired student t-test or two-way ANOVA followed by post-hoc multiple comparison (NS denotes no significance)

ANX-M1 administered before photo-oxidative damage showed no functional or morphological protection from damage in the retinas at day 14 compared to isotype control (Fig. 5). Retinal morphology (Fig. 5b-c), IBA1^+^ microglia/macrophage numbers (Fig. 5d) and ERGs (Fig. 5e-h) showed no significant differences between the treatment groups (*P* > 0.05; Fig. 5b-h), indicating that pre-damage treatment via intravitreal injection to neutralise C1q was ineffective.

### Systemic C1q inhibition using ANX-M1 anti-C1q antibody

**Fig. 6:**
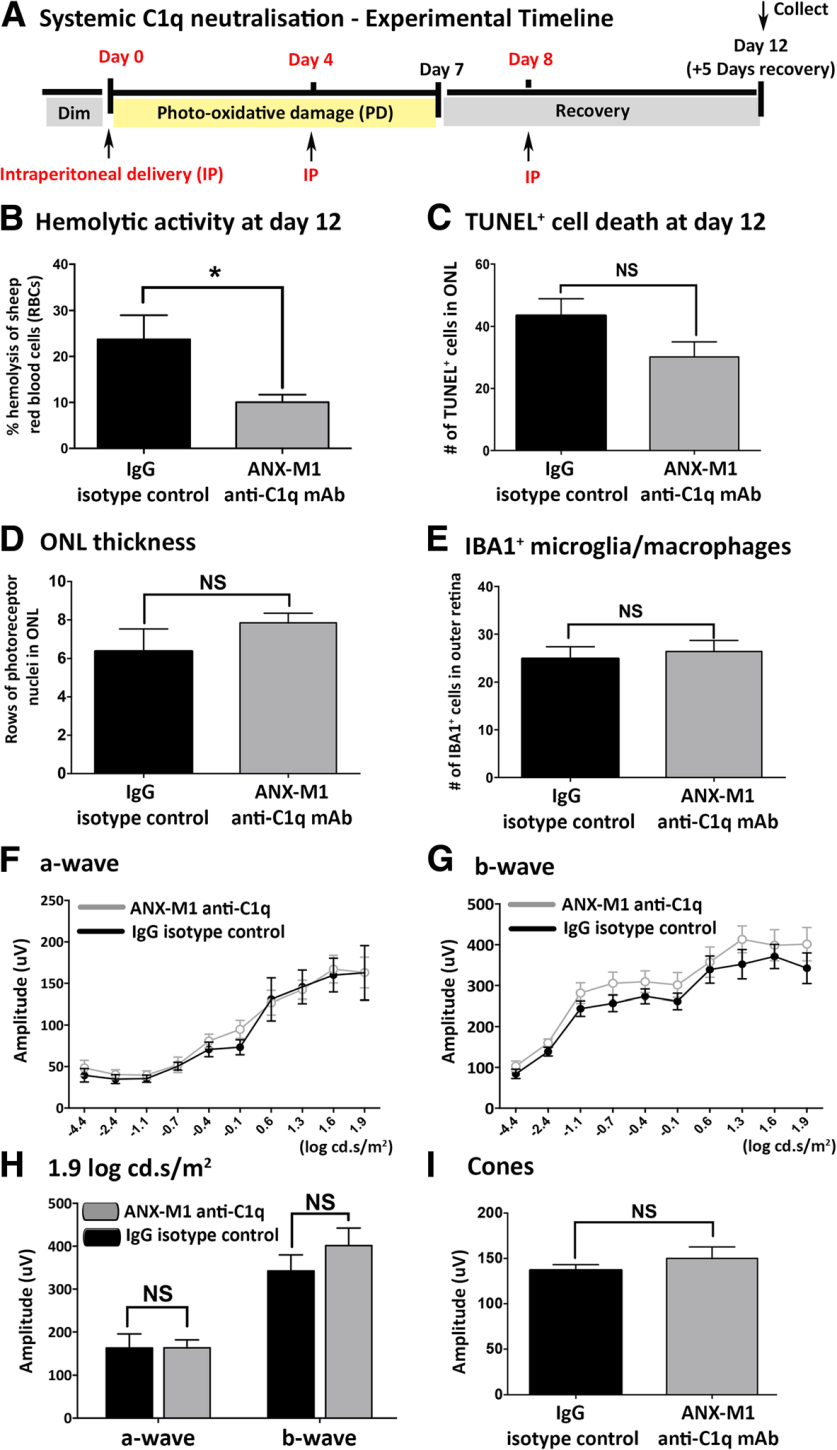
Neutralisation of systemic classical complement using ANX-M1 anti-C1q antibody during the time course of photo-oxidative damage (PD) and assessment of the retina at day 12 (**a**). **b** Complement activity in the serum was inhibited (> 50% less) in mice treated with ANX-M1 anti-C1q antibody compared to IgG control as examined in the hemolytic assay using sheep red blood cells (*P* < 0.05). **c-e** There was no difference in the number of TUNEL^+^ cells **(c)**, ONL thickness **(d)** and IBA1^+^ microglia/macrophages **(e)** observed between the ANX-M1 anti-C1q antibody cohort and IgG control cohort (*P* > 0.05). **f**-**h** Inhibition of systemic classical complement did not change retinal function, as evident in no significant difference in a-wave **(f)**, b-wave **(g)** response characteristics and mean amplitudes at highest flash intensities between the two cohorts **(h)** (*P* > 0.05). **i** No significant change in cone response was detected between the two cohorts (*P* > 0.05). Statistical analyses were determined by an unpaired student t-test or two-way ANOVA followed by post-hoc multiple comparison (*denotes *P* < 0.05; NS denotes no significance)

The results from animals treated systemically are presented in Fig. 6. A hemolytic assay using mouse serum from ANX-M1-treated and control mice showed that by day 12, serum complement activity was reduced by > 50% compared to the IgG control group, following the systemic delivery paradigm of ANX-M1 anti-C1q antibody (*P* < 0.05; Fig. 6b).

The impact of systemic C1q neutralisation on retinal morphology was not significant. No differences in the number of TUNEL^+^ cells, ONL thickness or IBA1^+^ microglia/macrophage numbers were observed in mice treated systemically with the ANX-M1 anti-C1q antibody (*P* > 0.05; Fig. 6c-e). Similarly, there were no significant functional differences in a-wave, b-wave or cone response at day 12, between the ANX-M1 anti-C1q antibody and IgG control antibody groups (*P* > 0.05; Fig. 6f-i).

### Inflammasome expression in *C1qa*^*−/−*^ mice

**Fig. 7:**
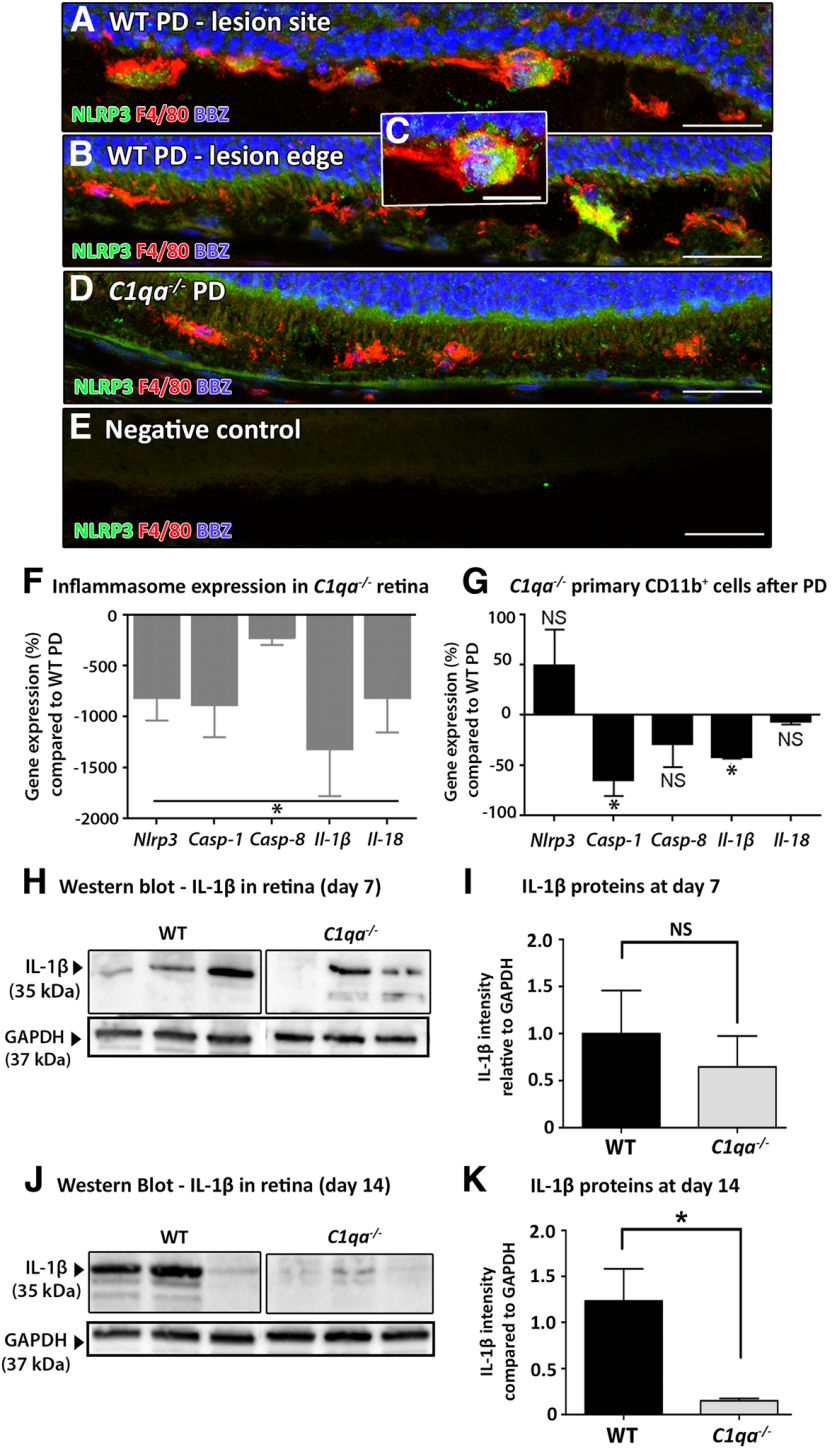
Inflammasome activation in wild-type (WT) and *C1qa*^−/−^ retinas. **a-b** NLRP3-expressing cells (green) localised with subretinal macrophages expressing F4/80 (red) in the subretinal space both at the lesion site and lesion edges at 7 days of photo-oxidative damage (PD). **c** NLRP3-expressing subretinal macrophages had NLRP3-immunopositive and bisbenzimide (BBZ)-immunopositive nuclei, confirming its localisation within the cell. **d** In the retina of *C1qa*^−/−^ mice, there were no NLRP3-expressing cells co-localising with F4/80^+^ macrophages. **e** Negative control omitting the primary antibodies displayed no specific staining for either NLRP3 or F4/80. **f** *C1qa*^*−/−*^ retina showed significantly reduced expression of inflammasome-related genes compared to WT following PD (*P* < 0.05). **g** Primary myeloid cells (CD11b^+^) isolated from *C1qa*^−/−^ retina by FACS had significantly reduced expression of *Il-1β* and *Casp-1* (*P* < 0.05), but not *Il-18, Nlrp3* or *Casp-8* (*P* > 0.05). **h**-**i** Western blots for IL-1β proteins at day 7 showed similar abundance of IL-1β proteins in *C1qa*^*−/−*^ and WT retinas; there was no significant change in the relative intensity of IL-1β protein bands between *C1qa*^*−/−*^ and WT retinas (*P* > 0.05). **j**-**k** At day 14, there was significantly less abundance of IL-1β proteins in *C1qa*^*−/−*^ retinas, compared to WT, as confirmed by densitometry (*P* < 0.05). Statistical analyses were performed using student t-test and one-way ANOVA followed by post-hoc multiple comparison (*N* = 4 per group; * represents *P* < 0.05; NS represents no significance). For representative images, scale bars represent 50 μm

To further assess the potential mechanism involved in modulation of the retinal inflammation observed in *C1qa*^*−/−*^ mice, we investigated the role of inflammasome activation in photo-oxidative damage. C1q is known to activate NLRP3 inflammasome and production of IL-1β in mouse bone-marrow derived macrophages (BMDMs) primed with carboxyethylpyrrole adducts (CEP), which are by-products of oxidative damage in dry AMD [21]. Immunolabelling of NLRP3 demonstrates co-localisation with F4/80^+^ macrophages in the subretinal space of WT mice following photo-oxidative damage (Fig. 7a-b). However, NLRP3^+^ or F4/80^+^ cells were not detected in the ONL, or in other retinal layers. The NLRP3^+^ F4/80^+^ macrophages detected in the subretinal space of WTanimals were distributed at the focal retinal lesion and towards the lesion edges, with the rounded amoeboid morphology of activated macrophages (Fig. 7a, c). No NLRP3-expressing cells were detected in the *C1qa*^*−/−*^ retinas, although F4/80^+^ macrophages were present in the subretinal space (Fig. 7d). A negative control that omitted the primary antibodies showed no specific staining for NLRP3 or F4/80 (Fig. 7e).

Expression of inflammasome-related genes was assessed in *C1qa*^*−/−*^ and WT retinas at 7 days of photo-oxidative damage, using qPCR. *Nlrp3*, *Casp-1* and *Casp-8* expression were significantly decreased in retinas of *C1qa*^−/−^ mice compared to WT at 7 days of photo-oxidative damage (*P* < 0.05, Fig. 7f). Additionally, expression of the pro-inflammatory cytokine genes *Il-1β* and *Il-18* were also significantly lower in *C1qa*^−/−^ mice compared to WT (~ 1500%, *Il-1β*; ~ 800%, *Il-18*; *P* < 0.05, Fig. 7f).

FACS isolation was used to analyse expression of inflammasome-related genes in retinal CD11b^+^ cells, isolated from *C1qa*^*−/−*^ and WT mice following photo-oxidative damage (Fig. 7g). Primary myeloid cells from *C1qa*^−/−^ retinas had a significantly reduced expression of *Il-1β* and *Casp-1* compared to WT retinas (*P* < 0.05, Fig. 7g), but there was no detectable change in the expression of *Il-18*, *Casp-8* or *Nlrp3* (*P* > 0.05, Fig. 7g).

The presence of IL-1β protein in *C1qa−/−* and WT retinas was used to confirm inflammasome expression at 7 days of photo-oxidative damage (day 7; Fig. 7h-i) and post-damage (day 14; Fig. 7j-k) using Western blot. There was no significant difference in the abundance of IL-1β proteins observed, between *C1qa*^*−/−*^ and WT retinas at day 7 (*P* > 0.05, Fig. 7i). At day 14 post-photo-oxidative damage, the IL-1β protein band in *C1qa*^*−/−*^ retinas was significantly less intense compared to retinas from WT mice (*P* < 0.05, Fig. 7k).

### Localisation of C1q in mouse and human AMD donor retinas

**Fig. 8:**
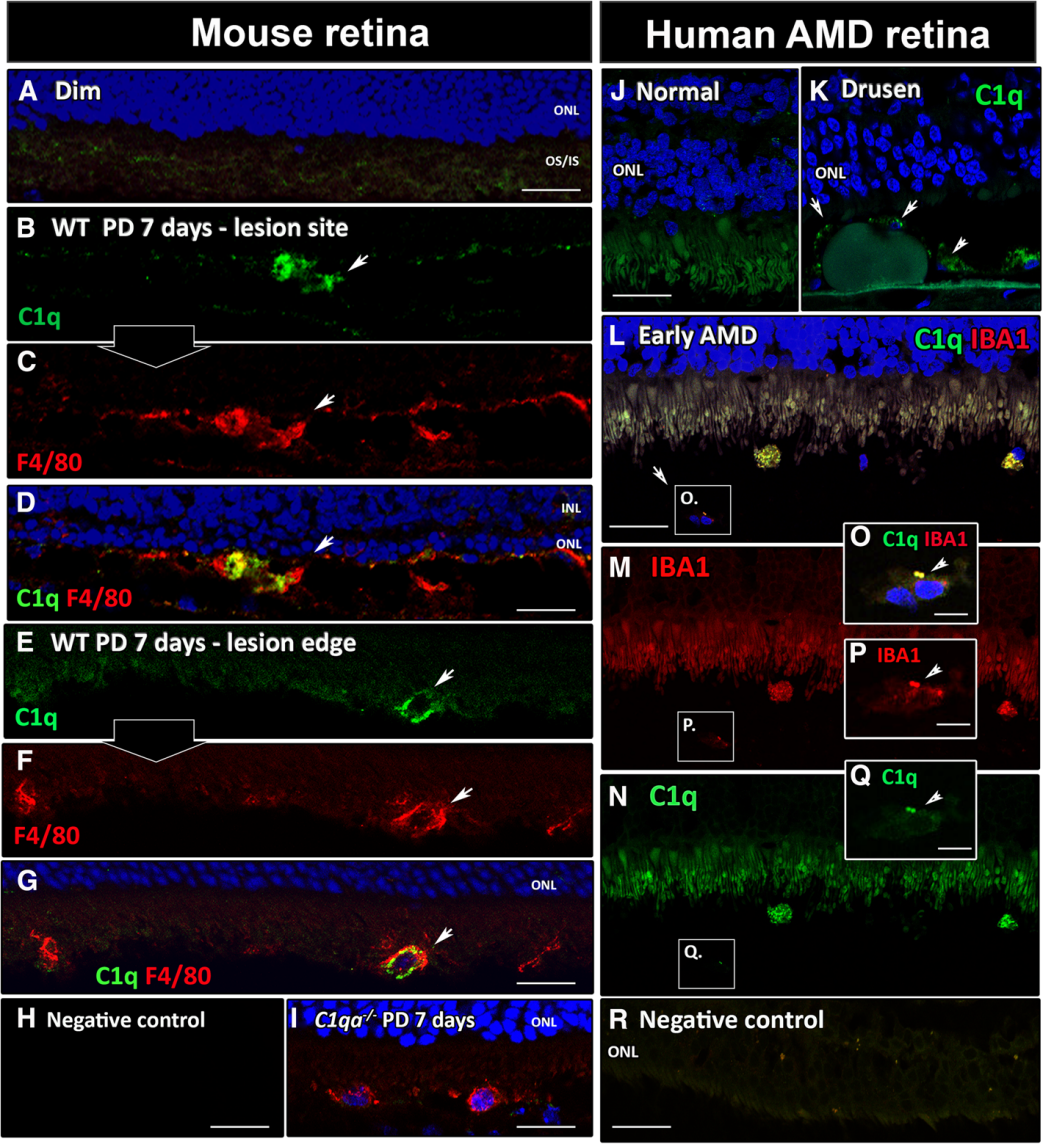
C1q localisation in the mouse retina after photo-oxidative damage (PD) and in human AMD retinas. **a** No immunoreactivity to C1q or F4/80 was detected in retinas from dim-reared/control animals. **b** At 7 days of PD, C1q^+^ cells were detected in the subretinal space at the lesion site. **c** At 7 days of PD, F4/80^+^ cells were also detected at the lesion site. **d** Co-localisation of C1q and F4/80 was observed in cells in the subretinal space at the lesion site. **e**-**g** At the lesion edge C1q^+^/F4/80^+^ macrophages were present in the subretinal space. **h** Negative control slide for the mouse retinal immunohistochemistry showed no specific staining for either C1q or F4/80. **i** There was no immunoreactivity for C1q protein in the retina of *C1qa*^*−/−*^ mice at 7 days after photo-oxidative damage. **j** C1q localisation in the normal human retina. No C1q-expressing cells were detected in the normal retina. **k** C1q immunoreactivity was detected adjacent to the subretinal drusen of early AMD retinas (arrows). **l**-**n** At the lesion edge, C1q-expressing cells were present in the subretinal space of AMD retina where IBA1^+^ cells were predominant (arrows). **o** C1q^+^ immunoreactivity co-localised in IBA1^+^ macrophages. **p, q** C1q^+^/IBA1^+^ cells at higher magnification. **r** Negative control slide for human AMD immunohistochemistry showed no specific staining for either C1q or IBA1. Representative images derived from *N* = 3 per group. INL, inner nuclear layer; ONL, outer nuclear layer; OS/IS, outer/inner segments. Scale bars represent 10 μm in O-Q; elsewhere 50 μm

In dim-reared mouse retinas (Fig. 8a-i), C1q protein or F4/80^+^ macrophages were not detected (Fig. 8a). In photo-oxidative damaged mouse retinas, C1q protein was co-localised with amoeboid, F4/80^+^ subretinal macrophages at the focal retinal lesion (Fig. 8b-d) and at the edges of the photoreceptor lesion (Fig. 8e-g). Negative controls showed no specific staining for either C1q or F4/80 in the mouse retinal sections (Fig. 8h). Absence of C1q immunoreactivity was confirmed in F4/80^+^ macrophages in the outer retina after 7 days of photo-oxidative damage in *C1qa*^*−/−*^ mice (Fig. 8i). Immunohistochemistry for the C1q protein in normal retina and AMD-affected retinas (Fig. 8j-r) showed no C1q-expressing cells in the normal retina (Fig. 8j). In the AMD-affected retinas, C1q proteins were detected adjacent to subretinal drusen (Fig. 8k), and in the subretinal space at the lesion edges (Fig. 8l-n). Double immunolabelling demonstrated that most of C1q-expressing cells were immunoreactive for macrophage/microglia marker IBA1 (Fig. 8o-q). Negative controls showed no specific staining for C1q or IBA1 in the human AMD section (Fig. 8r).

## Discussion

This study investigated the contribution of C1q and the classical complement pathway in the timing of disease progression induced in retinal degeneration. Our data illustrate the role of locally-derived retinal C1q in driving the progression of retinal atrophy and the possibility of therapeutic interventions using C1q neutralisation. The findings indicate that: ***First*,** genetic ablation of *C1qa* indicated variable and inconclusive effects on retinal function during the 7 days of photo-oxidative damage. At 1 week following photo-oxidative damage (day 14), the absence of C1q was found to protect retinal structure, reduce photoreceptor cell death and inflammation, and improve retinal function. ***Second***, we demonstrate a possible role for the classical complement pathway in the activation of the NLRP3 inflammasome and secretion of IL-1β in the degenerating retina, which may promote retinal damage during this post-exposure period. ***Third***, by comparing neutralization of classical complement activator C1q systemically and locally in the retina, we demonstrate that local C1q expressed by subretinal microglia/macrophages, and not serum complement, plays a vital role in the progression of retinal degeneration. Together, the results indicate that the classical complement pathway, mediated by C1q, contributes to progression of retinal degeneration. These results further indicate that a therapeutic approach targeting the classical complement initiator C1q may be beneficial in slowing the progression of focal retinal degenerations, where dysregulated complement is a major feature of disease pathogenesis, such as AMD.

### The classical pathway in retinal atrophy

C1q is associated with pathological features of age-related neurodegenerative diseases such as amyloid-β accumulation in Alzheimer’s Disease (AD), glaucomatous damage in a model of glaucoma and drusen deposition in AMD [37–39]. Evidence from other studies indicates that non-canonical functions of C1q influence local inflammation in the central nervous system (CNS), such as by promoting the dynamic shift of astrocytes to a more pro-inflammatory state which is associated with AD and Huntington’s Disease [40]. Moreover, C1q is involved in the elimination of synapses in a mouse model of AD, and blockade of C1q significantly preserved synaptic function [41, 42]. The ablation of *C1qa* further prevented the progressive loss of retinal ganglion cells in a mouse model of glaucoma [43]. However, a recent study demonstrated that numerous complement components, including C1q, were important in maintaining inner nuclear layer (INL) retinal integrity, and that the absence of C1q in the developing retina reduced retinal function in the aging retina [44]. Others have reported a neuroprotective influence of C1q on the survival of cone photoreceptors in *Rho−/−* mice and elucidated that C1q was involved in cone cell preservation in hereditary retinopathies [45], indicating a role of C1q in normal retinal development.

Our present findings suggest that C1q facilitates the progression of late-stage retinal atrophy, following the onset of retinal damage. Studies in our laboratory have previously shown upregulation of classical complement components *C1s, C4a* and *C2* in retinas following photo-oxidative damage [11], but their specific roles have not been described. Other studies on the pathological effects of *C1qa* in neurodegeneration have been implicated in mouse models of retinal ischemic injury [24], and glaucoma [43]. The findings presented here show that initially (days 1-7 of photo-oxidative damage) the absence of *C1qa* has no definitive impact on retinal degeneration induced by photo-oxidative damage; however, over an extended timeframe, sustained expression of *C1qa* is a driver of progressive loss of photoreceptors. Rohrer et al. (2010) suggest an involvement of the activation of the classical complement in the development of choroidal neovascularisation, in the late stage of AMD in a laser-induced rodent model of choroidal neovascularisation, since the alternative complement is not sufficient to produce the pathology [15]. Consistent with this concept, the data from the current study demonstrates that localised post-treatment with ANX-M1 C1q inhibition protects against retinal degeneration during the progression phase of damage (7-14 days), but not earlier during the onset of retinal degeneration. The findings suggest a potential role of C1q in mediating the progression of retinal degeneration.

### C1q and inflammasome activation by subretinal macrophages in photo-oxidative damage

C1q stimulation activates the NLRP3 inflammasome and the secretion of IL-1β in CEP-primed BMDMs [21]. NLRP3 inflammasome activation in AMD has been proposed to occur in response to several stimuli including drusen components, RPE and complement proteins, oxidative stress, oxidative by-products and DNA [21, 46, 47]. In this study, in the absence of classical pathway activation, there was a reduced NLRP3 inflammasome expression in a specific population of cells - the subretinal microglia/macrophages.

This finding is consistent with previous studies showing that C1q can activate NLRP3 in BMDMs of humans, and in mice after immunization with CEP-adducted mouse serum albumin, to model a dry AMD-like pathology [21]. Other evidence has demonstrated the capability of RPE cells to induce NLRP3 inflammasome expression and propagate photoreceptor toxicity in the presence of blue-light photo-oxidative damage [48]. We have previously shown that in photo-oxidative damage, inflammasome expression and IL-1β production were observed in retinal degeneration, where IL-1β was found to be expressed by microglia and macrophages at the site of photoreceptor degeneration [49]. Our findings from the current study show significant down-regulation of IL-1β in isolated retinal macrophages from *C1qa*^*−/−*^ retinas compared to wild-type controls, which correlated to a more preserved photoreceptor population at 14 days. The findings indicate that the reduced activity in the classical pathway dampens NLRP3 inflammasome expression along with pro-inflammatory IL-1β secretion, resulting in protection of the photoreceptor population.

It is of interest that we detected no significant difference in C3 expression by Western blot between WT and *C1qa*^*−/−*^ mice at day 14. This may indicate that the effector functions enabled by the classical complement pathway may be independent of hydrolysis of C3 [38, 50, 51], which is predominantly amplified by the alternative complement pathway [11] in photo-oxidative damage models of retinal degeneration. Our findings indicate that one of the non-canonical mechanisms of complement activation is associated with inflammasome activation, as indicated by decreased NLRP3 labelling in *C1qa*^*−/−*^ microglia. Further, C1q can trigger a rapid enhancement of phagocytosis upon recognition of apoptotic cells, independent of the classical component pathway activation [52]. For these reasons, we hypothesise that both inflammasome activation and enhanced phagocytosis are mediated by C1q, explaining why we see protection of retinal function in the *C1qa*^*−/−*^ mice, even though no changes in C3 were detected at 14 days. These findings highlight the intricate association between the classical pathway and inflammasome activation, although the precise role of C1q in inflammasome activation and other non-canonical functions, such as phagocytosis, warrants further investigation.

### Complement-expressing macrophages as potent local mediators of onset and progression of retinal atrophy

Retinal microglia/macrophages express complement components in rodent retinas in response to aging [31]. Here, we demonstrate that subretinal microglia/macrophages also express *C1q* in focal retinal degeneration*.* Our data show that subretinal microglia/macrophages in the degenerating retina can express CD68^+^ lysosomal protein aggregates, and contain multiple TUNEL^+^ apoptotic cells, demonstrating a phagocytic phenotype. It is likely that C1q expression may be found in these phagocytic macrophages of the subretinal space, as we have detected C1q expression co-localising with subretinal microglia/macrophages in the degenerating retina after photo-oxidative damage. It is plausible that C1q is secreted to bind and recognize defective synapses at a stage of disease prior to overt neurodegeneration, as reported in models of neurodegenerative diseases in the brain and retina [38, 43], however this was not observed directly in this study.

In normal and diseased brain tissues resident microglia express many-fold higher levels of *C1qa*, *−b* and *–c*, the three protein subunits of C1q, compared to macrophages and other myeloid cells [53, 54]. The capacity of microglia to synthesize *C1qa* has been confirmed in rodent brain [55], however, in the context of AMD the close interdependence and proximity of the retina with Bruch’s membrane/RPE/choroid confounds the argument that microglia are the key synthesizers of C1q. Our findings demonstrate that C1q proteins are localised near drusen and in the subretinal microglia/macrophages of human AMD patients whose RPE atrophy was prominent. The delayed appearance of these C1q-expressing subretinal microglia/macrophages, coincides with RPE and choriocapillaris breakdown [56] suggesting a population derived from the choriocapillaris. However, a recent study investigating models of RPE breakdown suggests that subretinal populations are predominantly resident microglia, owing to their rapid mobilisation from the inner retina to the site of injury [57].

Our findings with the local delivery of the C1q neutralisation suggest that locally-derived C1q contributes to the progression of retinal atrophy. Coupled with the association of C1q expressed by myeloid cells with neuroinflammation and age-related neurodegeneration [58], our findings support the idea that locally administered immunotherapy targeting the classical complement pathway may be effective in slowing the progression of retinal degeneration. We did not detect any ocular inflammation specifically associated with anti-C1q delivery in these experiments. This suggests that administration of an anti-C1q antibody is unlikely to have additional risks to those normally associated with intravitreal injections (reviewed in [59]). Further investigations are required to explore the mechanisms underlying the protective effect of local C1q neutralisation.

## Conclusions

This study illustrates the contribution of the classical complement pathway in facilitating retinal atrophy induced by photo-oxidative damage. The classical pathway, through its initiating molecule C1q*,* appears to exacerbate photoreceptor damage through interaction with the inflammasome. The efficacy of intravitreal C1q-targeted inhibition in ameliorating the late-stage of photo-oxidative damage-induced retinal atrophy, reinforces the concept that targeting C1q, or the macrophage capability to express complement, may be effective in slowing the progression of retinal degenerations. Further utilisation of strategies that can elucidate the exact origins of complement-expressing macrophages - microglia or monocytes - are warranted. Clarifying the identity and function of these complement-expressing myeloid cells will enable the design of more precise therapeutics targeting the complement system, which may be beneficial for retinal diseases where macrophage recruitment and complement activation are a key pathogenic feature, including AMD.

## Additional files

Additional file 1: **Figure S1.** Characterisation of dim-reared C57BL/6 J wild-type (WT) and *C1qa*^*−/−*^ mouse retinas. A-C: The ERG a-wave and b-wave responses also did not change significantly between WT and *C1qa*^*−/−*^; cone response also displayed no difference between two groups (*P* > 0.05). D: No difference was shown in the expression of neuroprotective genes (*P* > 0.05) except for *Fgf2*, which showed a significantly downregulation compared to WT (*P* < 0.05; *N* = 5 animals per group). Statistical significance was determined by an unpaired student t-test or two-way ANOVA with multiple comparison post hoc (* represents *P* < 0.05; NS represents no significance). (TIF 8158 kb)

Additional file 2: **Figure S2.** Retinal C3d protein levels in wild-type (WT) dim-reared controls and after 7 days of photo-oxidative damage (PD). A: C3d protein levels significantly increased in retinas following 7 days of photo-oxidative damage (*P* < 0.05; *N* = 4). C3d levels were normalised to a GAPDH loading control. B-C: Representative western blots showing an increased level of C3d after 7 days of photo-oxidative damage (C) compared to dim-reared controls (B). Statistical significance was determined by an unpaired student t-test (* represents *P* < 0.05). (TIF 4344 kb)

## Abbreviations

ACTB: beta-actin
AD: Alzheimer’s Disease
AMD: Age-Related Macular Degeneration
ANOVA: Analysis of variance
ANU: Australian National University
ARVO: Association for Research in Vision and Ophthalmology
BBZ: Bisbenzimide
BMDM: Bone-marrow derived macrophage
C1q: Complement component 1q
C1qa: Complement component 1q, subcomponent alpha
C1s: Complement component 1 s
C2: Complement component 2
C3: Complement component 3
C3d: Complement component 3d
C4: Complement component 4
CASP-1: Caspase 1
CASP-8: Caspase 8
CD11b: Cluster of differentiation molecule 11b
cDNA: Complementary DNA
CEP: Carboxyethylpyrrole
CFB: Complement factor B
CFH: Complement factor H
CFI: Complement factor I
CO_2_: Carbon dioxide
DNA: Deoxyribonucleic acid
dUTP: 2′-deoxyuridine 5′-triphosphate
ERG: Electroretinography
FACS: Fluorescence-activated cell sorting
GAPDH: Glyceraldehyde-3-phosphate dehydrogenase
GWAS: Genome-wide association studies
I/R: Ischemia/reperfusion
IBA-1: Ionised calcium binding adaptor molecule 1
IgG: Immunoglobulin G
IL-18: Interleukin 18
IL-1β: Interleukin 1β
INL: Inner nuclear layer
IS: Inner segments
IVC: Individual ventilated cage
LED: Light-emitting diode
mAb: Monoclonal antibody
MAC: Membrane attack complex
MBL: Mannose-binding lectin
NLRP3: NOD-like receptor protein 3
ONL: Outer nuclear layer
OS: Outer segments
P: Postnatal day
PBS: Phosphate-buffered saline
PCR: Polymerase chain reaction
PD: Photo-oxidative damage
PE: Phycoerythrin
qPCR: Quantitative real-time PCR
RNA: Ribonucleic acid
RPE: Retinal pigment epithelium
SERPING1: Serpin family G member 1
TUNEL: Terminal deoxynucleotidyl transferase (TdT) dUTP nick end labelling
WT: Wild-type
ΔΔC_t_: Comparative cycle threshold
