# Supplementary figures and images for: Subretinal macrophages produce classical complement activator C1q leading to the progression of focal retinal degeneration
Source: Mol Neurodegener. 2018 Aug 20;13:45. doi: 10.1186/s13024-018-0278-0 (PMC6102844; doi:10.1186/s13024-018-0278-0)

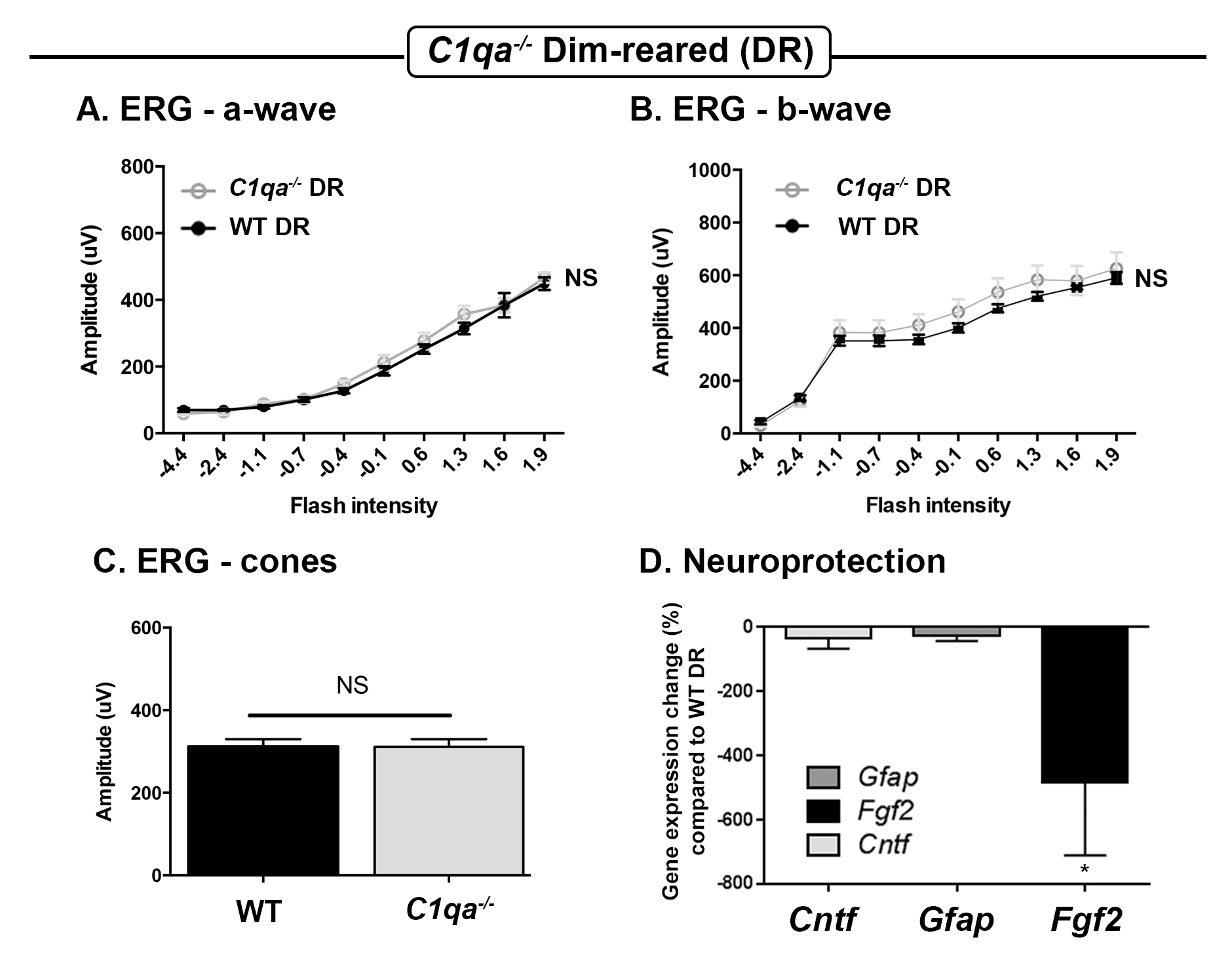

Supplement: Supplementary file 1 — Figure S1. Characterisation of dim-reared C57BL/6 J wild-type (WT) and C1qa−/− mouse retinas. A-C: The ERG a-wave and b-wave responses also did not change significantly between WT and C1qa−/−; cone response also displayed no difference between two groups (P > 0.05). D: No difference was shown in the expression of neuroprotective genes (P > 0.05) except for Fgf2, which showed a significantly downregulation compared to WT (P < 0.05; N = 5 animals per group). Statistical significance was determined by an unpaired student t-test or two-way ANOVA with multiple comparison post hoc (* represents P < 0.05; NS represents no significance). (TIF 8158 kb) [file 13024_2018_278_MOESM1_ESM.tif]

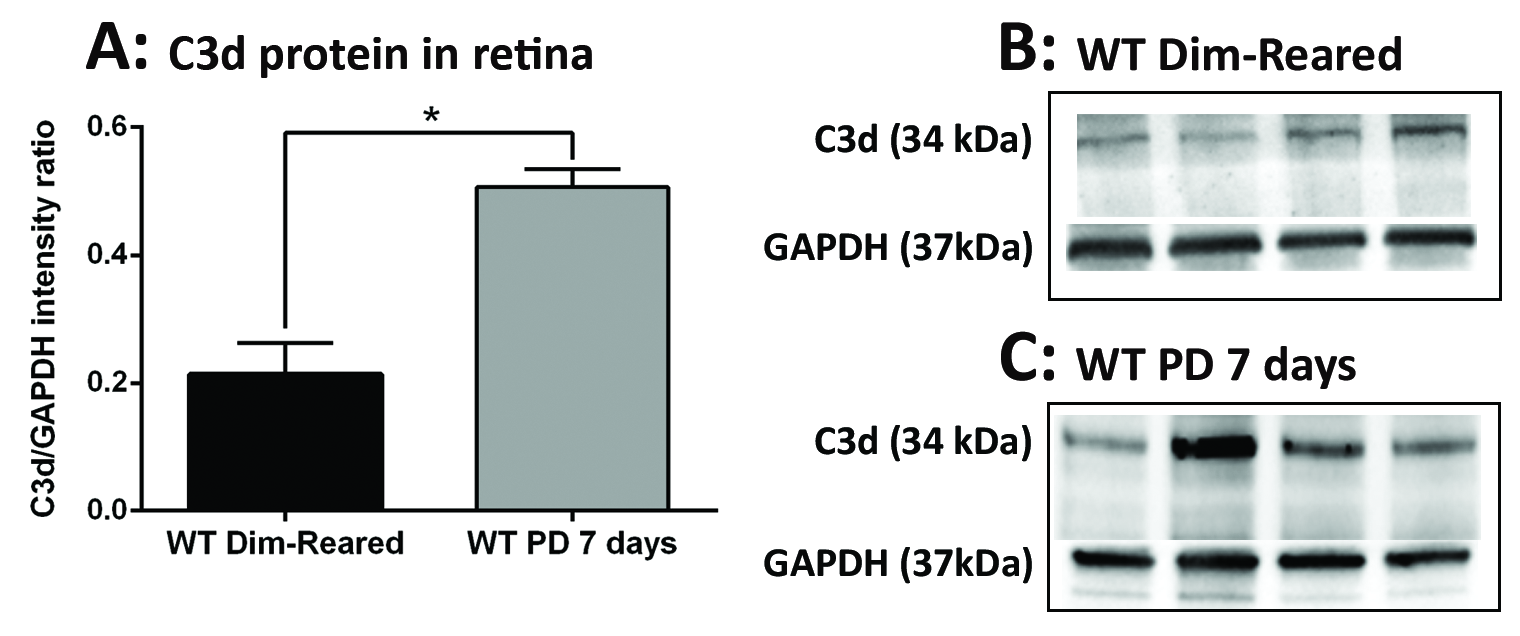

Supplement: Supplementary file 2 — Figure S2. Retinal C3d protein levels in wild-type (WT) dim-reared controls and after 7 days of photo-oxidative damage (PD). A: C3d protein levels significantly increased in retinas following 7 days of photo-oxidative damage (P < 0.05; N = 4). C3d levels were normalised to a GAPDH loading control. B-C: Representative western blots showing an increased level of C3d after 7 days of photo-oxidative damage (C) compared to dim-reared controls (B). Statistical significance was determined by an unpaired student t-test (* represents P < 0.05). (TIF 4344 kb) [file 13024_2018_278_MOESM2_ESM.tif]
